# On line clinical reasoning assessment with Script Concordance test in urology: results of a French pilot study

**DOI:** 10.1186/1472-6920-6-45

**Published:** 2006-08-28

**Authors:** Louis Sibert, Stefan J Darmoni, Badisse Dahamna, Marie-France Hellot, Jacques Weber, Bernard Charlin

**Affiliations:** 1 Department of Urology, Rouen University Hospital, 1, rue de Germont 76031 Rouen Cedex, France; 2 CISMeF, Rouen University Hospital, France & GCSIS, LITIS Lab, Rouen University, France, CISMeF & GCSIS, 1, rue de Germont, 76031 Rouen Cedex, France; 3 Department of Biostatistics, Rouen University Hospital, 1, rue de Germont, 76031 Rouen Cedex, France; 4 Department of Medical Education, Rouen University Hospital, 1, rue de Germont, 76031 Rouen Cedex, France; 5 Unit for Research and Development in Health Sciences Education, Faculté de Médecine-Direction, University of Montreal, CP 6128, succursale centre-ville, Montreal, Quebec H3C 3J7, Canada

## Abstract

**Background:**

The Script Concordance test (SC) test is an assessment tool that measures the capacity to solve ill-defined problems, that is, reasoning in a context of uncertainty. This study assesses the feasibility, reliability and validity of the SC test made available on the Web to French urologists.

**Methods:**

A 97 items SC test was developed based on major educational objectives of French urology training programmes. A secure Web site was created with two sequential modules: a) The first one for the reference panel to elaborate the scoring system; b) The second for candidates with different levels of experience in urology: Board certified urologists, chief-residents, residents, medical students. All participants were recruited on a voluntary basis. Statistical analysis included descriptive statistics of the participants' scores and factorial analysis of variance (ANOVA) to study differences between groups' means. Reliability was evaluated with Cronbach's alpha coefficient.

**Results:**

The on line SC test has been operational since June 2004. Twenty-six faculty members constituted the reference panel. During the following 10 months, 207 participants took the test online (124 urologists, 29 chief-residents, 38 residents, 16 students). No technical problem was encountered. Forty-five percent of the participants completed the test partially only. Differences between the means scores for the 4 groups were statistically significant (*P *= 0.0123). The Bonferroni post-hoc correction indicated that significant differences were present between students and chief-residents, between students and urologists. There were no differences between chief-residents and urologists. Reliability coefficient was 0.734 for the total group of participants.

**Conclusion:**

Feasibility of Web-based SC test was proved successful by the large number of participants who participated in a few months. This Web site has permitted to quickly confirm reliability of the SC test and develop strategy to improve construct validity of the test when applied in the field of urology. Nevertheless, optimisation of the SC test content, with a smaller number of items will be necessary. Virtual medical education initiative such as this SC test delivered on the Internet warrants consideration in the current context of national pre-residency certification examination in France.

## Background

The main aim of medical education is to foster the development of clinical competence in students at all levels. As in any health profession, clinical competence comprises of a number of dimensions. Although a sound knowledge base, clinical and interpersonal skills are vital for a physician; clinical reasoning represents a major component of clinical competence. A significant part of clinical reasoning rests on the capacity of applying well-known solutions from Evidence-Based Medicine (EBM) in defined and stable contexts. However, the usefulness of applying EBM to individual patient is limited [1]. Reasoning in the medical profession is much more than simple applications of knowledge, rules and principles. Individual clinical expertise relies on the capacity to deal with uncertainty. In a clinical encounter, not all the data required to solve a problem are available. These data must be retrieved in order to formulate the problem and then solve it. Furthermore, problems can be confusing, contradictory and ill defined [2], and are often characterized by imperfect, inconsistent or even inaccurate information. The capacity to reason in the context of uncertainty and to solve poorly defined problems is a hallmark of professional competence in medicine.

Traditional tools for assessing clinical reasoning, such as rich-context, multiple choice questions (MCQ) [3], extended matching questions (EMQ) [4] correctly and reliably test the ability of students to apply well-known solutions from EBM to well defined problems. Test formats based on written simulations of clinical problem solving have repeatedly shown the puzzling fact that experienced clinicians judged competent by peers, often perform slightly better, and sometimes worse than clinicians with intermediate levels of experience [3]. Other important limitations of this type of assessment are difficulties of standardization, objectivity of scoring, and practicability for large groups of examinees. A further difficulty with assessment on ill-defined problems is that, as shown in medicine, in similar situations professionals do not collect the exact same data and do not follow the same reasoning patterns [5]. They also show substantial variation in performance as regards any particular real or simulated case [6]. Furthermore, most current performance-based methods of professional competence assessment (e.g. Objective Structured Clinical Exams) [7,8] are only measures of observable clinical skills.

At a time when cognitive psychology has become the major conceptual framework in the education of professions [9], the adaptation of script theory [10,11] to the characteristics of reasoning in the health professions provides a promising way to build a theory-based assessment tool. This theory implies that in order to give meaning and to act effectively to a given situation, professionals activate goal-directed knowledge structures relevant to the situation. These structures, named scripts, are used to actively process information to confirm or eliminate hypotheses, or management options [11]. Based on this theory, reasoning is performed with a series of qualitative judgments. Each of these judgments can be measured and compared to those of a reference panel of experienced practitioners. This provides a method of assessment for reasoning on ill-defined problems and in contexts of uncertainty [12,13]. This method is called the script concordance approach.

The approach is based on three principles, each concern one of the following three components [14] inherent to any test: 1) the task required of examinees represents an authentic clinical situation and is described in a vignette. This vignette does not contain all the data required to provide a solution and several options (diagnosis, management or attitude) should be considered. 2) The response format is in accordance with what is known based on the clinical reasoning process [5,6]. A Likert scale, measuring the judgments that are constantly made within this process, retrieves examinees' answers. 3) The scoring method takes into account variation of answers among jury members. Credits on each item are derived from the answers supplied by a panel of reference. The method to build the tool is described in detail later in the article.

When developing any type of assessment, there are 3 criteria that must be taken into account: validity, reliability and practicability [3,4]. Studies on the SC test have consistently shown a linear increase in the mean scores of individuals with different increased levels of clinical expertise; the less experienced getting the lower results. These findings support the construct validity of the instrument [13,15]. A study was carried out to verify whether scores obtained on a SC test taken at the end of clerkship predict those obtained on tests of clinical reasoning (written simulations) 2 years later at the end of residency. Data found support the predictive validity of the test [16]. An other study found that the scoring method provided higher scores to tested experts and allowed a better discrimination of scores among examinees, suggesting the validity of the scoring process [17]. Further research findings indicate that the script concordance approach may permit testing in domains that have hardly been assessed to date, such as perception and interpretation skills in film reading [18] and difficult therapeutic situations that implicate ethical judgment [19]. As regards reliability, a test is often considered to be sufficiently reliable when its Cronbach's alpha coefficient reaches a value of .80. In published series, values ranged from 0.79 to 0.82. [12,13,15-18]. Experience shows that an 80-item test can be taken in 1 hour or less. This compares very favourably with the time required by other examination formats to reach the .80 values. Finally, research findings have shown that SC test has another advantage for a testing method of being relatively easy to construct and to administer [12,13,15,17,18].

Nevertheless, experience of SC test on a large population remains limited. No research, to our knowledge, has yet been conducted throughout an entire country. Further extensive research is still required to verify psychometric characteristics and to assess the educational impact of the test in our learning environment. Its diffusion on a large scale should permit to confirm its utility as a strategy for investigating the process of decision making within the health professions. Furthermore, information and communication technologies (ICT) are gradually becoming a central part of medical education; in particular, the French Medical Virtual University consortium [20,21] of 29 French medical schools out of 32 has the objective of sharing experiences throughout the country using ICT to support new pedagogical approaches for medical students. In this context, we have recently introduced a Web site to promote an on line assessment course of clinical decision-making in context of uncertainty with the SC test, in order to allow administration of SC test on a very large number of candidates. Initial results on the feasibility of this web-based SC test, conducted in the field of urology, were previously reported in the BMC Medical Informatics and Decision Making journal [22].

The goal of this paper was to report the results of the first on line large-scale utilisation of the SC test in the field of urology. The study explored the psychometric properties of the SC test when delivered on the Web to French urologists within the first year of utilisation. In order to answer the research question, participants were recruited from the entire country and were identified according to their level of experience in urology (board certified urologists, chief-residents, residents and students).

## Methods

### Development of the SC test

A bank of SC test items for urology has been developed since May 2001 by researchers from the Rouen University Hospital and the Faculty of Medicine of the University of Montreal (LS and BC) according to the methodology previously described [12,13]. Two faculty members were asked to a) describe clinical situations representative of urology practice and based on major educational objectives of urology training programmes; b) specify for each situation, the questions they would ask and the actions they would take to arrive at a diagnosis or decide on the adequate management of the patient. Test items were built using the material obtained at this stage.

The clinical situations are described in short vignettes. The description of the situation must be complex enough to be challenging for the level of training that has to be assessed (urology residency, in this context). They must not contain all the data to provide a unique solution. Each vignette is followed by a series of related items. The item format differs with the objective of assessment (diagnosis, investigation, or treatment). For a given vignette, items are regrouped by formats (e.g. some items on diagnosis, followed by some items on investigation). Each item consists of three parts. The first part includes a diagnostic hypothesis, an investigative action or a treatment option. The second presents new information (e.g. a clinical data, imaging study or laboratory test result) that might have an effect on the diagnostic hypothesis, the investigative action or treatment option. The third part is a 5-point Likert-type scale that records the participant answer (see illustration of the 3 formats in Table 1). Each item was built so that a reflection was necessary to answer it. It was also clearly specified in the instructions for each participant that within the vignettes, each item is independent of the others. Hypotheses or options change for each question. An example of items from the therapeutic section of the test is illustrated in Table 2. A total of 115 SC test items from the bank were included in the database and constituted the SC test on line.

### Development of the Web site

The development process of the Web site was described in our preliminary report [22]. This process adheres to the main principles of practical guidelines for developing effective educational website [23] and is based on the traditional three-tier architecture used in Web database applications.

### Scoring process

The scoring process is derived from the aggregate scoring method [24], following the common methodology used in the SC test [12,13]. This method takes into account the variability experienced clinicians demonstrate in their reasoning processes. Credits on each item are derived from the answers given by a panel of reference. For each item, candidates' answers received a credit mark corresponding to the proportion of panel members who selected it. The maximum score for each item was 1 for the modal answer. Other panel members' choices received a partial credit. Answers not chosen by panel members received 0. To obtain this proportional transformation, the number of panel members who had provided an answer on the Likert scale was divided by the modal value for the item. For example, if on an item, six panel members (out of 10) have chosen response +1, this choice receives a score of 1 point (6/6). If three panel members chose response +2, this choice receives a score of 0.5 (3/6), and if one panel member chose response 0, this choice receives a score of 0.16 point (1/6). The total score for the test is the sum of credits obtained on each item, which in the end was transformed to obtain a maximum of 100.

### Participants

#### Reference panel

Reference panel included a relatively broad sample of experienced urologists with variability in demographics, training background and level of experience, thus constituting an appropriate population for development of a norm referenced database of performance and for study of the assessment method. An information seminar regarding the SC test on line was presented during the Annual Seminar of the French Society of Urology. All members of the Society were invited to participate on a voluntary basis. Previous studies have demonstrated that recruiting more than 10 members in a reference panel presents a reliable assessment of clinical reasoning with the SC test and using more than 20 members shows only a marginal benefit in terms of psychometrics properties [13,17]. Taking into account theses data, minimum expected number of participants for the reference panel was 20. They were asked to fill out the test online individually, exactly as performed by the candidates. After their completion of the test, members of reference panel were asked to identify the items they found confusing or not relevant. Items that generated unimodal or bimodal experts' responses on the Likert scale were also discarded. In total, 18 items were then excluded. Final SC test submitted on line to candidates was made up of 97 items and 17 clinical situations.

#### Candidates

In order to assess psychometric properties of the SC test, participants were identified according to their level of experience in urology. Four groups of participants with different levels of experience in urology were recruited during the same seminar than members of reference panel: Board certified urologists, chief-residents, residents, and medical students (5 or 6^th ^years). All agreed. Inclusion criteria to participate in the SC test were: for the Board certified Urologists, to be member of the French Urological Association (AFU) with an access to the SC test on line via the AFU Web site [25]; for the urology residents, to be trainees of the national urology training programme; and for the medical students, to have a rotation in urology during the past six months before the SC test. In order to provide a robust statistical analysis in a faster period of time, minimum expected numbers of participants were 100 for the board certified urologists, 80 for the total of chief-residents and residents and 50 for the medical students.

### Statistical analysis

Item scores and total scores for each participant were computed and statistical analyses were performed using the SAS software (SAS Institute Inc., Cary, North Carolina, Version 8.0). Descriptive statistics of the participants' scores on the SC test were performed, followed by an ANOVA to evaluate differences between the group's mean scores. To evaluate the presence of a significant statistical difference, a *P *< 0.05 was considered as significant. A Bonferroni correction procedure was then used to precisely determine which score differences were significant between groups of participants. With this correction, 6 outcomes were tested: urologists vs. chief-residents, urologists vs. residents, urologists vs. students, chief-residents vs. residents, chief-residents vs. students, and residents vs. students. To evaluate the presence of a significant statistical difference, an adjusted *P *< 0.05/6 = 0.0083 was considered as significant. Reliability of the examination was assessed via the Cronbach's alpha internal consistency coefficient. In this study, the items were used as units of reliability analysis, in order to be representative of reliability measures used in previous published studies on SC test

## Results

**Figure 1:**
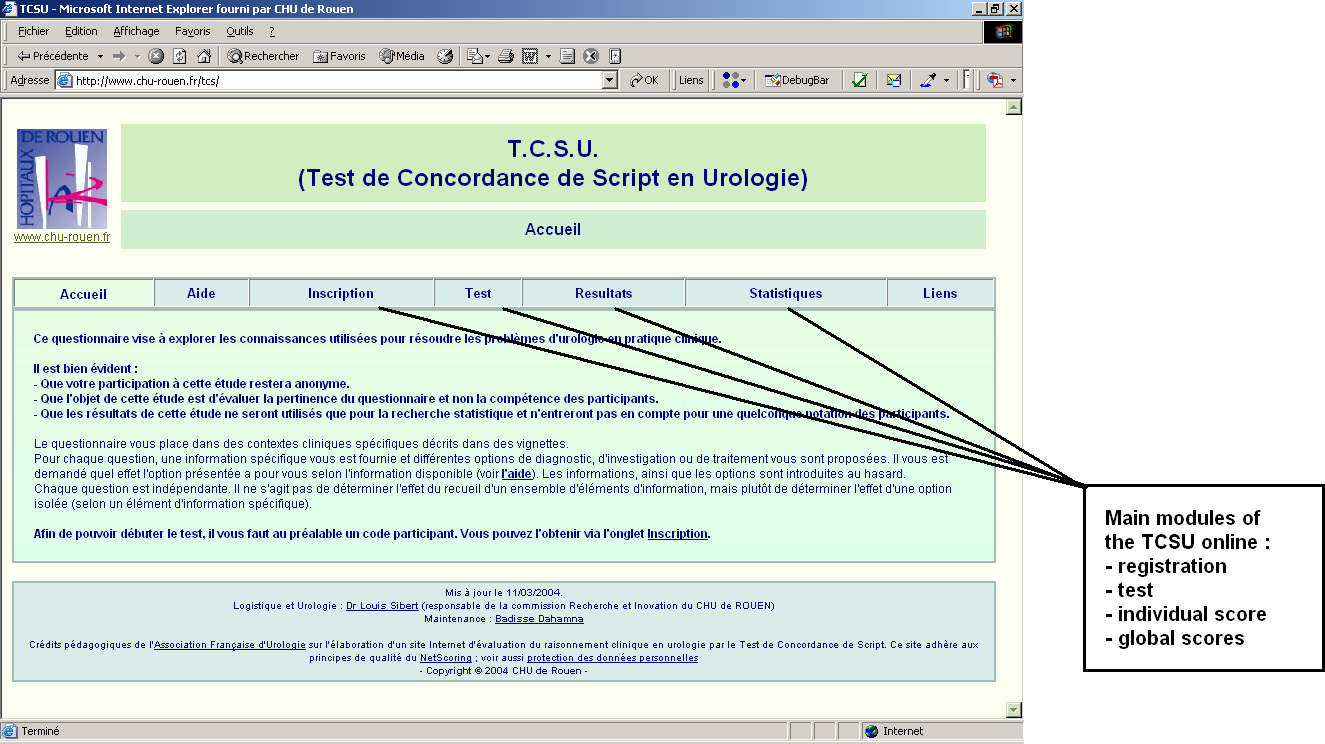
Illustration of the SC test on line home page (URL: ).

The SC test was placed on line on the Web site of Rouen University Hospital, which is 24/7 since its creation in February 1995 [26]. A secure Web site was created for the two populations (reference panel and candidates). The evaluation system can be accessed via any computer system with a standard Web browser. A userid/password is required for each individual to enter the "SC test online" Web site. A second userid/password is necessary for the reference panel. The SC test online home page (see Figure 1) contains several modules: one to register as a candidate or as a member of the reference panel, another to pass the test, another to obtain the individual test score for each candidate and another to obtain the global scores by groups or by demographic data. The home page contains a summary of the SC test principles as well as the instructions for the participants.

The SC test on line has been operational since June 2004. A total of 26 urologists from University, general and private practice completed the SC test during the following two months and constituted the reference panel. The panel members completed their test in less than 70 minutes. The SC test on line was subsequently available to the participants in September 2004. Ten months later, 207 candidates had already passed the SC test on line: 124 urologists, 29 chief-residents, 38 residents and 16 medical students. During this period, no technical problems were encountered on the web site. Two e-mails were sent due to difficulties in subscription (over 233 participants). Nevertheless, approximately 45% of the participants did not answer all the items. In cases where the participant did not complete the SC test, he was not given a score. A total of 61 urologists (49%), 15 chief-residents (50%), 27 residents (70%) and 10 students (60%) fully completed the test (113). The statistical analysis was performed on these 113 participants.

Global mean scores for the different groups of participants according to their level of experience in urology are summarised in Table 3. The mean scores were 69.91 ± 6.72 for the urologists, 70.50 ± 4.92 for the chief-residents, 68.49 ± 6.01 for the residents, and 63.12 ± 4.61 for the students. The urologists showed the widest range of scores (33.76), followed by residents (25.73), chief-residents (17.47) and students (13.55). Differences between the mean scores for the four groups were considered statistically significant (*P *= 0.0123). The post-hoc Bonferroni analysis indicated that significant differences were present between students and chief-residents (*P *= 0.0025) and between students and urologists (*P *= 0.0005). Differences were present between students and residents but there were not statistically significant. There were no differences between chief-residents and urologists. The Cronbach's alpha reliability coefficient for the test was 0.734.

## Discussion

The major goal of this study was to determine psychometric characteristics of the SC test on line. As regards validity, our results showed that students obtained significant lower results than chief-residents and than urologists. Students performance was lower than residents performance but without any statistical difference. The lack of significance in the difference of scores between the other participants' groups can be explained by the higher variability of residents and urologists' scores. Despite the global correlation of scores with level of training observed in our study, the construct validity of the SC test on line is not straightforward. Nevertheless, it is interesting to note that the less experienced group of participants obtained the lower results. The reliability of the SC test on line appears to be satisfactory with a Cronbach's alpha coefficient value of 0.734 for the total group of participants. Feasibility of the SC test on line has been previously established [22]. Furthermore, practicing urologists and residents appear to enjoy completing a test that is close to real clinical reasoning, as demonstrated by the numbers of urologists, chief-residents and residents who performed the SC test via our Web site despite their lack of experience of on line evaluation systems.

Despite the exploratory format of this study, our results are well correlated with other studies on SC test carried out in different medical specialties, which have previously demonstrated the discriminant validity and the reliability of the SC test [12,13,15-19]. Sample size of participants and reference panel of our study were comparable to those described in the literature. Furthermore, previously reported SC test administrations were paper-based. Many researches have demonstrated that widely used paper-based evaluation systems are costly and time-consuming, with difficulty in data analysis and significant delays in identifying problem trends [4]. The present study now provides useful issues concerning the potential of Web-based SC test. Our Web site has provided a cost-effective opportunity to include participants without logistic difficulties traditionally encountered with paper-based evaluation systems in organizing the entire reference panel, chief-residents, residents and students' meetings. Furthermore, in our experience among French urological community [15], we learnt that organizing traditional face-to-face teaching and evaluation sessions is difficult with such a large number of dispersed busy practitioners. Considering how often it is difficult to recruit examination jury members [12,13], the feasibility of recruiting more than 20 members on the reference panel via our Web site, underlines the utility of the Internet for the development of assessment course in medical school curricula. The SC test on line has the additional advantage of access from an off-site location at any time and place convenient to the urologists and other participants.

Several other Web sites for SC tests are also currently being developed in Bordeaux, France and in Montreal, Canada [27]. The Bordeaux Web site is focusing on CME [28]. To our knowledge, only one other study regarding SC test on line, performed in neurosurgery was recently published, but with no statistical analysis [29]. Montreal, Bordeaux and Rouen, are currently building a consortium to promote on line clinical reasoning assessment using SC tests for national examinations.

Our findings have several limitations. The first is the lower number of participants than expected. In particularly, only 32% of the target figures for the students performed the SC test on line. Therefore, there is a possible lack of power in the statistical analysis to detect any significance in the difference of the scores between participating groups. The lack of difference in the scores between chief-residents and urologists could be partially explained by the fact that chief-residents involved in this study were at the end of their curricula and most of the urologists included were recently board certified. Therefore, clinical experience of the two groups could be considered more or less similar. Feedback possibilities of our Web site should permit us to focus on the recruitment of more students.

The other limit of our methodology is that only 55% of the participants have entirely completed the SC test. Based on this result, the fact that participants never received e-mail reminders during the inclusion period should be taken into consideration. Nevertheless, improvement in the completion rate of SC test on line will be necessary for future research. To address this issue, it would be possible to organized training sessions using video conferencing to teach participants how to log on to the system and complete the SC test. The high number of test questions (97) could also probably explain the rate of completion. Previous studies have repeatedly demonstrated that a reliability coefficient value of 0.80 could be reached (or almost reached) with 60 to 70 items [12,13,15-19]. An optimisation process of SC test content with a smaller number of items is currently under development. This Web site also presents some technical limitations, i.e. multimedia resources (sounds, images, and videos), which were not previously developed. In fact, the aim of our study was to include a maximum number of participants via the Internet. These new functionalities will be implemented in the last semester of 2006 and will enhance the differences between the SC test on paper vs. the SC test on the Internet. Finally, any given examination instrument has its limitations. Professional competence in medicine is a multi-dimensional entity and cannot be adequately measured by a single assessment method. SC test should be used in complement with what could be considered as the optimal tool of evidence-based factual knowledge assessment, i.e. rich-context MCQ as used by the National Board of Medical Examination in the United States [30]. These concepts underline the current need to promote this type of Web site.

Although our study is limited in several ways, interesting perspectives could be obtained from our exploratory research. Efficient and meaningful evaluation of clinical competence is currently critical of professional development of trainees in medical training programmes. ICT now offer the possibility to validate learning and assessment tools on a large scale over a relatively short period of time: i.e. 100% of the Board certified urologists and 83% of the expected number of the chief-residents and residents performed the SC test on line during a ten month period. The technical concept of the SC test on line enabled an automatic auto evaluation of the results immediately after the online examination. In addition to being able to inform participants of their results in real time, it will be also possible to reduce correction time and personnel resources. The facility of the Internet to implement SC test on line is obvious. One can imagine that test modules will be available on electronic campuses. The physician would be able to select any module, pass the SC test, and either succeeds (sufficiently high score when compared with the reference panel) and obtains training credits or fails. In this case the physician may receive a notification of the areas of weakness with hyperlinks to relevant references. The physician can then later carry out training specifically focused on areas of weakness.

The Script Concordance approach is designed to measure the quality of a set of cognitive operations or knowledge structures by comparing a participant's problem representation, judgements and choices to those of an experienced clinicians group. The test can be used in situations where there is no consensus among experts, in daily medical practice. Finally, SC test offers the opportunity of a wide range of assessment of decision-making skills in contexts where evidence-based medicine cannot be applied. Therefore, with the use of the Internet, this should facilitate a more accurate approach regarding the utility of this tool. For instance, the educational impact of the SC test in the overall urology training programmes is currently under study, since it was integrated this year into the continuing medical education of the AFU Web site.

## Conclusion

In practice, the number of participants recruited over a short period of time in this study has encouraged us to extend this experiment to other medical disciplines, including therapeutics. The Rouen University Medical School is one of the founders of the FMVU consortium. One of the FMVU aims is to adapt "SC test on line" for formative evaluations of clinical reasoning to prepare medical students to the new pre-residency examination in France, which is written simulation-based. In the near future, SC test may be included as a part of this national summative examination, which has a very large audience (n = 4,000 students). An on line SC test prototype was recently designed and is now freely accessible [31]. In this context, virtual medical education initiative such as this Web site warrants further consideration.

## Competing interests

The author(s) declare that they have no competing interests.

## Authors' contributions

LS was the main investigator of the project and drafted the manuscript. SJD and BD developed the Web site, were responsible for data acquisition and revised the manuscript. MFH performed the statistical analysis. Critical revisions were carried out by JW. BC was the inventor of the SC test, had the original idea of on line assessment of clinical reasoning with the SC test. He was involved in conceiving and planning the study and revised the manuscript. Funding was obtained by LS. All authors have read and approved the final manuscript.

## Pre-publication history

The pre-publication history for this paper can be accessed here:



## Figures and Tables

**Table 1:** Illustration of questions and answering grid format.

For diagnostic knowledge assessment		
If you were thinking of	And then you find	This hypothesis becomes
(*A diagnosis hypothesis*)	(*A new clinical information, an imaging study or a laboratory test result*)	-2 -1 0 +1 +2
-2 the hypothesis is almost eliminated		
-1 the hypothesis becomes less probable		
0 the information has no effect on the hypothesis		
+1 the hypothesis is becoming more probable		
+2 it can only be this hypothesis		


For investigation knowledge assessment		
If you were considering to ask	And then you find	This investigation becomes
(*A diagnostic test*)	(*A new clinical information, an imaging study or a laboratory test result*)	-2 -1 0 +1 +2
-2 contra-indicated totally or almost totally		
-1 not useful or even detrimental		
0 nor less nor more useful		
+1 useful		
+2 absolutely necessary		


For treatment knowledge assessment		
If you were considering to prescribe	And then you find	The relevance of this treatment becomes
(*A therapeutic option*)	(*A new clinical information, an imaging study or a laboratory test result*)	-2 -1 0 +1 +2
-2 contra-indicated totally or almost totally		
-1 not useful or even detrimental		
0 nor less nor more useful		
+1 useful		
+2 necessary or absolutely necessary		

The item format varies with the object of assessment (e.g. diagnostic, investigation, treatment).

**Table 2:** Example of a clinical vignette with items from the therapeutic section of the SC test.

If you were considering doing:	And then you find:	The effect on the relevance of this treatment becomes:
Abstention and surveillance	Almost normal clinical examination, but presence of a microscopic hematuria	-2 -1 0 +1 +2
Surgical exploration	Normal clinical examination, despite an aching epididymitis point	-2 -1 0 +1 +2
Abstention and surveillance	The trauma occurred 3 days ago and clinical examination reveal inflammatory scrotum	-2 -1 0 +1 +2
Surgical exploration	Normal clinical examination. A scrotal ultrasonography shows a hematocele layer and an intra-testicular contusion with no apparent fracture	-2 -1 0 +1 +2

A 27 year-old male patient has been admitted in the emergency room for a scrotal trauma during a football match. The emergency doctor contacts you for this patient's therapeutic care.

**Table 3:** Comparison of mean scores by groups according to level of clinical experience in urology.

**Group**	**Size**	**Mean score ± SD**	**Minimum**	**Maximum**
**Urologists**	61	69.91 ± 6.72	50.51	84.27
**Chief-residents**	15	70.51 ± 4.92	60.93	78.40
**Residents**	27	68.49 ± 6.01	50.31	76.04
**Students**	10	63.12 ± 4.61	53.44	68.09

- Statistical analyses were performed only on the 113 participants out of 207 who fully completed the test.

- SD: standard deviation

- ANOVA: *P *= 0.0123

- Bonferroni correction analysis: Students vs. Chief-residents, *P *= 0.0025; Students vs. Urologists: *P *= 0.0005. Other comparisons between groups were not significant.
